# Multiple long-range host shifts of major *Wolbachia* supergroups infecting arthropods

**DOI:** 10.1038/s41598-022-12299-x

**Published:** 2022-05-17

**Authors:** Tiago M. F. F. Gomes, Gabriel L. Wallau, Elgion L. S. Loreto

**Affiliations:** 1 Programa de Pós-Graduação em Genética e Biologia Molecular, Universidade Federal do Rio Grande do Sul, Porto Alegre, Rio Grande do Sul Brazil; 2 Departamento de Entomologia, Instituto Aggeu Magalhães, Fundação Oswaldo Cruz, Recife, Pernambuco Brazil; 3 Biochemistry and Molecular Biology Department, Federal University of Santa Maria, Av. Roraima 1000, Santa Maria, RS CEP 97105.900 Brazil

**Keywords:** Coevolution, Evolutionary genetics, Phylogenetics, Population genetics

## Abstract

*Wolbachia* is a genus of intracellular bacterial endosymbionts found in 20–66% of all insect species and a range of other invertebrates. It is classified as a single species, *Wolbachia pipientis*, divided into supergroups A to U, with supergroups A and B infecting arthropods exclusively. *Wolbachia* is transmitted mainly via vertical transmission through female oocytes, but can also be transmitted across different taxa by host shift (HS): the direct transmission of *Wolbachia* cells between organisms without involving vertically transmitted gametic cells. To assess the HS contribution, we recovered 50 orthologous genes from over 1000 *Wolbachia* genomes, reconstructed their phylogeny and calculated gene similarity. Of 15 supergroup A *Wolbachia* lineages, 10 have similarities ranging from 95 to 99.9%, while their hosts’ similarities are around 60 to 80%. For supergroup B, four out of eight lineages, which infect diverse and distantly-related organisms such as Acari, Hemiptera and Diptera, showed similarities from 93 to 97%. These results show that *Wolbachia* genomes have a much higher similarity when compared to their hosts’ genes, which is a major indicator of HS. Our comparative genomic analysis suggests that, at least for supergroups A and B, HS is more frequent than expected, occurring even between distantly-related species.

## Introduction

*Wolbachia* is a genus of gram-negative intracellular endosymbiotic bacteria. First isolated from *Culex pipiens*, it is currently estimated to be found in 20–66% of all insect species^1^. Moreover, it also infects species of filarial nematodes, arachnids, and terrestrial crustaceans^2^. *Wolbachia* belongs to the Rickettisiales order, the same order of vertebrate pathogens transmitted by arthropod vectors, although there is no evidence of *Wolbachia* causing disease in vertebrates^3,4^. There are a myriad of *Wolbachia* lineages that differ substantially at the genomic level, but they are all classified under the umbrella of a single species *Wolbachia pipientis.* Its strains are divided into supergroups, ranging from A to U, which are defined by phylogenetic analysis using the 16S rDNA, *ftsZ* and *wsp* markers^5^. It is estimated that these supergroups diverged around 100 million years ago, first in filarial nematodes and then infecting arthropods. The supergroups A and B have only been found in arthropods so far; the C and D supergroups are specific to filarial nematodes; and the E and F supergroups are mostly found in nematodes, but are also seen in some terrestrial arthropods. The remaining supergroups are distributed among other arthropod clades^6^.

Long-term evolution of *Wolbachia* and their hosts have driven the emergence of diverse ecological relationships from mutualism to parasitism, depending on the lineage/supergroup-host pair. Parasitic *Wolbachia* lineages modulate different aspects of host physiology, such as the reproductive cycle, host behaviour and pathogen susceptibility^1,7^. Nematode-infecting *Wolbachia* usually have a mutualistic association with their hosts, whereas arthropod-infecting *Wolbachia* are more associated with commensalism or parasitism, modulating their host reproductive system through male-killing, feminization, parthenogenesis or cytoplasmic incompatibility^8^. The variety of *Wolbachia* induced phenotypes on their hosts has attracted the attention of the scientific community due to its potential role in host speciation, exploitation as a biological tool of vector-borne diseases control (e.g*.*, dengue, malaria), and to combat filarial neglected tropical diseases^9^.

*Wolbachia* is transmitted mainly via vertical transmission, i.e., it is passed between host generations in the female oocytes^10^. *Wolbachia* is also transmitted to other individuals and species through an alternative mechanism called host shift (HS), also referred as horizontal transfer, which is the direct transmission of *Wolbachia* cells between organisms where there is no feasible mechanism of vertical transfer.

HS can alter host fitness by adding phenotypes to the new host that allow it to interact most successfully with the environment^11^. *Wolbachia* strains that can manipulate the host reproductive biology achieve a high rate of infection in the new host, substantially enhancing *Wolbachia* spreading in the next host generation^6^.

As an obligatory endosymbiont that is mainly vertically transmitted, *Wolbachia* is expected to share a long evolutionary journey with their hosts. Nevertheless, there is strong evidence of *Wolbachia* ancient and recent horizontal transfer events between phylogenetically closely and distantly related host species^12–16^. Transfection experiments of *Wolbachia* were able to show its great capability to infect cells from distantly-related hosts, reinforcing the HS potential of *Wolbachia*^7,17,18^. Other characteristics that may influence HS include the ability of *Wolbachia* to survive for months in an extracellular environment, despite being an intracellular symbiont^6^, as well as genome recombination, which may influence the ability of the bacterium to adapt to new environments due to genome diversification^7^.

Despite the strong evidence on *Wolbachia* HS in several arthropod hosts, it is still considered a rare phenomenon^19,20^. In this study, we leveraged a large dataset of over 1000 draft and complete *Wolbachia* genomes reconstructed by Scholz et al. performing the most extensive assessment of *Wolbachia* HS so far. Our in-depth investigation of *Wolbachia*-host gene divergence revealed several long-range *Wolbachia* HS events from supergroups A and B among arthropods, suggesting HS is more frequent than normally reported for these abundant and widespread supergroups.

## Materials and methods

### Data

Assembled *Wolbachia* genomes were downloaded in November 2020, from https://www.ebi.ac.uk/ena/browser/view/PRJEB35167^21^; only *Wolbachia* genomes belonging to supergroups A and B were kept for analysis. Scholz retrieved existing *Wolbachia* reference genomes from refseq^22^ and genbank^23^, and public shotgun sequencing samples were retrieved from the NCBI sequence read archive (sra) database from all available projects involving taxa that can host *Wolbachia*. Host genomes were downloaded from https://www.ncbi.nlm.nih.gov/genome using the host species as a query term. The complete list of hosts and *Wolbachia* assemblies can be seen in Supplementary Table 1.

### Orthologue identification

The orthologous genes for both *Wolbachia* and their hosts were obtained using the BUSCO v5.1.2 docker image^24^ using the ‘augustus’ flag. The databases used were ricketisialles_odb10 and arthropoda_odb10 for *Wolbachia* and hosts, respectively. Fifty single-copy genes (Supplementary Table 1) for each strain were extracted from both searches for supergroups A and B, and single-copy genes shared between supergroups A and B to build a single evolutionary *Wolbachia* tree. In both situations, BUSCO was not able to recover 50 single-copy orthologues between all strains, the mean of recovered genes was 48.94 genes, standard deviation of 3.83 approximately. In those cases, the maximum possible number of genes for each strain was used.

### Alignment

Each one of the recovered orthologous genes were codon aligned separately using MACSE v2.05^25^, using the ‘alignSequences’ option, then all genes were concatenated by fasta identifier (ID) using the tool catfasta2phyml (available at https://github.com/nylander/catfasta2phyml) generating one fasta file with all sequences for hosts and *Wolbachia*, respectively.

### Similarity analysis and descriptive statistics

The command-line tool CIAlign^26^, version 1.0.9, was used to calculate the similarity between the concatenated aligned *Wolbachia* sequences, as well as for the host’s aligned sequences, using the following options: ‘--make_similarity_matrix_input’, ‘--make_simmatrix_keepgaps 2’. All descriptive statistics were calculated using the ‘describe’ method from the Python package Pandas. The ‘described’ method was also used to obtain the overall descriptive statistics for the mean, minimum and maximum values of the first generated statistics. The code used is available at https://github.com/Tiago-Minuzzi/wolbachia-hs.

### Phylogenetic analysis

The software IQ-Tree stable release 1.6.12^27^ was used to obtain the *Wolbachia* phylogeny, with the ultrafast bootstrap parameter set to 1000 and model GTR + F + R3 chosen according to BIC; the ITOL web server^28^ was used to generate the tree visualisation.

## Results

### Phylogenetic reconstruction and lineages

*Wolbachia* assemblies were separated into supergroups based on the phylogeny by Scholz et al. A careful assessment of the alignments revealed many identical sequences between different *Wolbachia* assemblies, thus, the fasta IDs of the identical sequences were grouped, and only a single sequence was kept as a representative. After selection of representative sequences, a reduction of 1044 to 304 sequences occurred for supergroup A and from 20 to 17 for supergroup B. Most of these highly similar genomes were characterised from different populations of some model organisms, such as species from the *Drosophila* genus.

**Figure 1:**
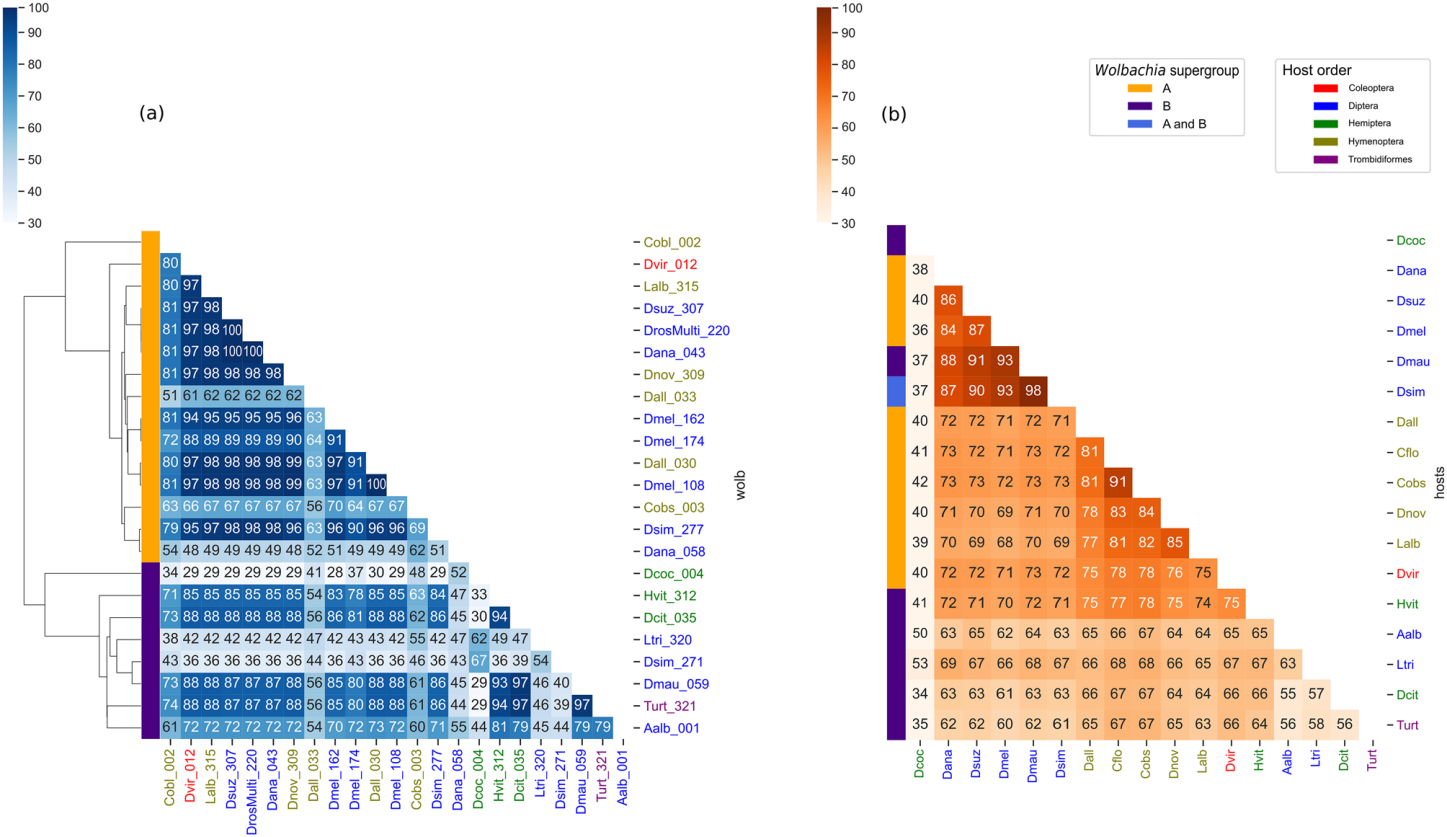
Heatmap showing: (**a**) *Wolbachia* similarity and (**b**) hosts similarity. *Wolbachia* heatmap shows the similarity from representatives of clades from supergroups A and B, also showing the *Wolbachia* phylogeny.

We reconstructed the *Wolbachia* phylogeny using 50 single-copy orthologues for both supergroups A and B to evaluate if the resulting tree agrees with the original dataset from Scholz et al. and showed that it matched as expected. After reconstructing the *Wolbachia* phylogeny, we grouped sequences in 23 lineages/clades that showed divergence lower than 0.02% (Supplementary Fig. 1), followed by random selection of one sequence from each *Wolbachia* lineage to estimate and compare the similarities between lineages (Fig. 1).

Supergroup A is composed of 15 lineages, occurring in 10 different hosts species. It is important to highlight that 10 out of these 15 lineages have similarities ranging from 95 to 99.9%, occurring in eight different host species, some of them as evolutionarily distant as Hymenoptera, Coleoptera and Diptera. These groups showed lower genetic similarity, ranging from 60 to 80% when comparing host genes (Fig. 1b). Supergroup B is composed of eight lineages found in eight different hosts. Four of these species belonging to distantly related taxa such as Acari, Hemiptera and Diptera showed *Wolbachia* gene similarities ranging from 93 to 97%. The graphical representation of gene alignments for *Wolbachia* supergroup A and supergroup B, and their hosts (Supplementary Figs. 2, 3; Supplementary Tables 2, 3) shows the high similarity within each *Wolbachia* supergroup and the lower similarity of host genes.

### Pairwise gene sequence similarity

**Figure 2:**
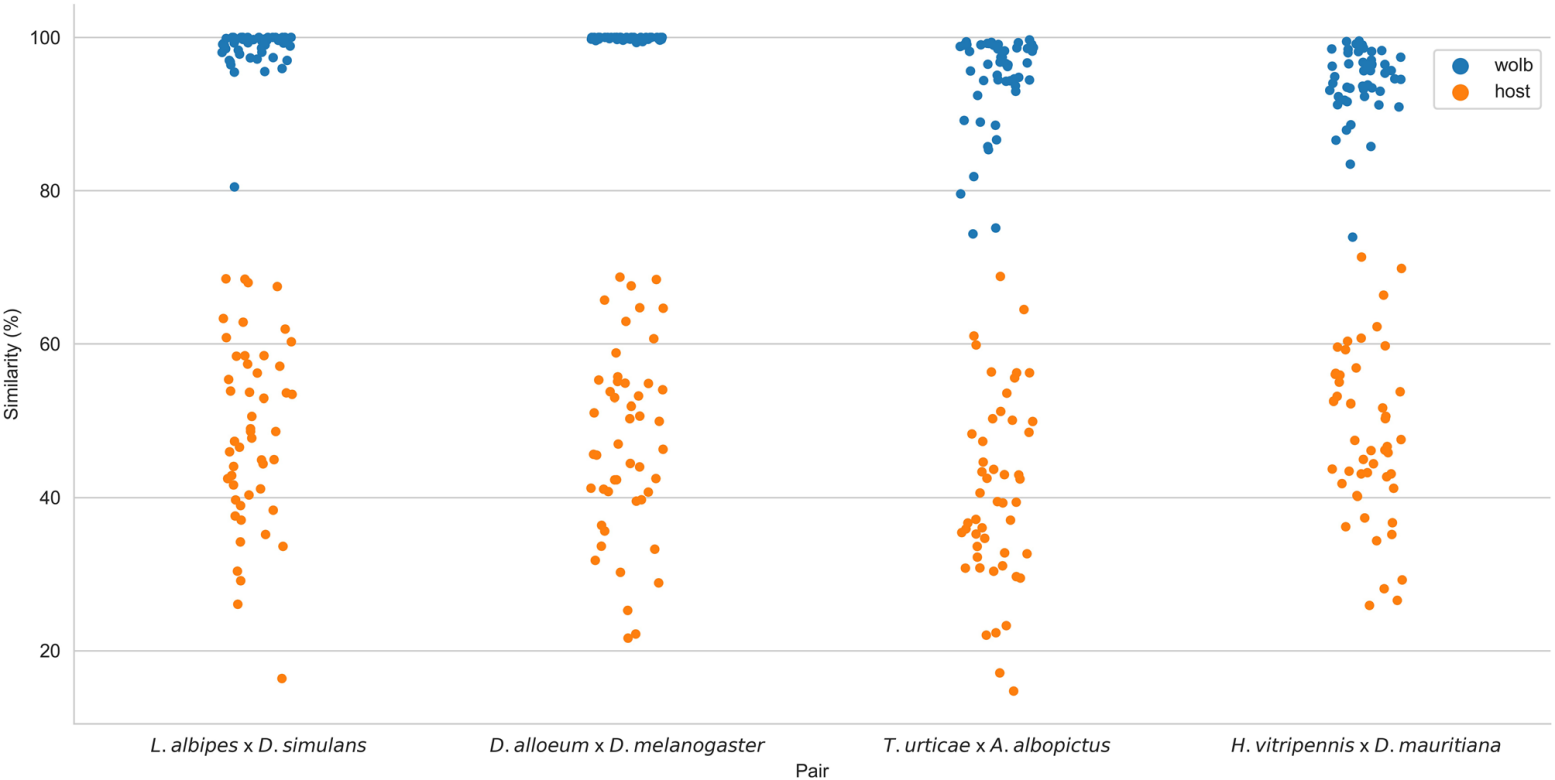
Pairwise gene similarity of *Wolbachia* and hosts. Each dot represents a gene pair (blue—*Wolbachia* genes; orange—host genes). It shows a higher similarity of *Wolbachia* orthologues when compared with their hosts orthologues similarity.

Pairwise gene sequence similarity analysis (Table 1) of *Wolbachia* and host orthologues shows striking differences (Fig. 2), corroborating the concatenated divergence analysis shown in Fig. 1. For supergroup A, the mean similarity between the Hymenopteran *Lasioglossum albipes Wolbachia* (assembly WOLB0007) and dipteran *Drosophila simulans Wolbachia* (WOLB0926) orthologues was 98.51% (minimum 80.48% and maximum 99.9%); the similarity between host orthologues was 48.36% (minimum similarity 16.37% and maximum similarity 68.49%). For *Wolbachia* of Hymenopteran *Diachasma alloeum* and dipteran *Drosophila melanogaster*, WOLB1002 and WOLB0092, respectively, the mean similarity was 99.87% (minimum 99.33% and maximum similarity 99.9%); host mean similarity values were 47.24% (minimum and maximum values, 21.64% and 68.71%, respectively).

**Table 1:** Descriptive statistics of pairwise gene sequence similarity of *Wolbachia* and hosts.

	Supergroup A				Supergroup B			
	*L. albipes *vs.* D. simulans*		*D. alloeum *vs.* D. melanogaster*		*T. urticae *vs.* A. albopictus*		*H. vitripennis *vs.* D. mauritiana*	
	Host	Wolb	Host	Wolb	Host	Wolb	Host	Wolb
n_genes	50	50	48	47	50	50	48	50
Mean	48.36%	98.51%	47.24%	99.87%	40.80%	94.37%	47.81%	94.17%
Std	11.77%	2.92%	12.21%	0.16%	12.07%	6.27%	10.84%	4.71%
Min	16.37%	80.48%	21.64%	99.33%	14.73%	74.35%	25.91%	73.93%
25%	40.52%	98.05%	40.45%	99.77%	32.68%	93.83%	41.66%	92.46%
50%	48.18%	99.42%	46.63%	99.99%	39.42%	96.54%	46.42%	95.10%
75%	57.29%	99.99%	54.93%	99.99%	49.57%	98.78%	55.95%	97.32%
Max	68.49%	99.99%	68.71%	99.99%	68.80%	99.67%	71.33%	99.53%

*n_genes* number of genes, *std* standard deviation, *min* minimum value found, *max* maximum value found.

For supergroup B, the *Wolbachia* found infecting the arachnid *Tetranychus urticae* (WOLB0958) and the strain infecting the insect *Aedes albopictus Wolbachia* (WOLB1128) showed a mean orthologue similarity of 94.37% (minimum 74.35% and maximum 99.67%), while the similarity between host orthologues showed a 40.80% mean similarity (minimum 14.73% and maximum 60.80%). The mean similarity for *Wolbachia* orthologues of the hemipteran *Homalodisca vitripennis* and dipteran *Drosophila mauritiana*, assemblies WOLB0957 and WOLB0080, respectively*,* was 94.17% (minimum 73.93% and maximum 99.53%); the mean similarity for host orthologues was 47.81% (minimum 25.91% and maximum 71.33%). To more clearly visualise the differences between the hosts and bacteria orthologous gene divergences, they are presented as strip plots for four pair-wise species comparisons (Fig. 2). Considering that *Wolbachia* mutation follow their hosts’ molecular clock, as demonstrated by the correlation of *Wolbachia* and the 18S rRNA gene evolution^21^, we can directly compare the evolution through time of *Wolbachia* and host genes, which demonstrates that the host genes are significantly more divergent than the *Wolbachia* genes.

### Supergroup A overall similarity

From the supergroup A similarity table (Supplementary Table 2), we calculated the descriptive statistical values for *Wolbachia* similarity within the supergroup for the following examples. *Wolbachia* from *Diabrotica virgifera*, order Coleoptera, showed a mean similarity of 84.38% with the Wolbachia from *Diachasma alloeum*, order Hymenoptera, with a maximum mean of 97%, a minimum mean of 60.15%, and a mode of maximum values of 97.19% (Supplementary Table 5). In *D. virgifera* and *Drosophila melanogaster* (Diptera) *Wolbachia,* the mean similarity, in many cases, is greater than 96%, reaching maximum values greater than 97%, with an overall mean similarity of 89.08%, mode of maximum values of 97.2% in a comparison of 150 *D. melanogaster* and 22 *D. virgifera Wolbachia* (Supplementary Table 6)*. D. virgifera Wolbachia* has an overall mean similarity of 96.05% with *Dufourea novaeangliae* (Hymenoptera) *Wolbachia* (Supplementary Table 7). The overall mean similarity between *D. virgifera* and *Drosophila ananassae Wolbachia*, is 90.37%, with a mean of max values of 90.9%, and a mode of max values of 96.40% (Supplementary Table 8).

### Supergroup B overall similarity

In Supergroup B, orthologue similarity analysis (Supplementary Table 3) and descriptive statistics (Supplementary Table 9) show that *Wolbachia* from Hemiptera *Diaphorina citri* has a mean similarity of 93.88% with *Wolbachia* from *Tetranychus urticae*, order Trombidiformes, Class Arachnida (minimum 88.56% and maximum 95.67%). The *D.* citri and *Drosophila mauritiana Wolbachia* similarity was 93.04% (minimum 88.19% and maximum 94.7%); *D. citri Wolbachia* similarity with *Wolbachia* from *Homalodisca vitripennis* (Hemiptera) was 93.49% (minimum 88.34% and maximum 95.25%); and *D. citri Wolbachia* has a mean similarity of 90.92% with *Wolbachia* from *A. albopictus* (minimum 86.45% and maximum 93.08%).

## Discussion

*Wolbachia* is the most widespread endosymbiotic organism in arthropods. One of the main features thought to be responsible for its successful long-term persistence in nature is its ability to manipulate host physiology and specifically host reproductive biology, conferring fitness benefits to *Wolbachia* and eventually to its host, including, for instance, increased pathogen resistance^29^. Maternal transmission, or vertical transfer, is the main process used by *Wolbachia* to infect a new host offspring, which, through evolutionary time, may allow these bacteria to prevail in different host species. Additionally, *Wolbachia* infection also can occur via hybridisation and introgression of similarly related species, or by HS between closely and distantly related species^30^.

Although *Wolbachia* HS is a well-documented phenomenon^6,7,18,31–34^, a large amount of the literature depicts it as a rare event^19,20^. Our comparative genomic analyses of several *Wolbachia* strains and their hosts reinforce the occurrence of HS in these bacteria, showing many cases in which different host species share *Wolbachia* more similar than would be expected by long-term coevolution of vertically transmitted endosymbionts with their hosts. However, the novel finding of our data is that HS, at least for *Wolbachia* supergroups A and B, seems to be more frequent than expected.

**Figure 3:**
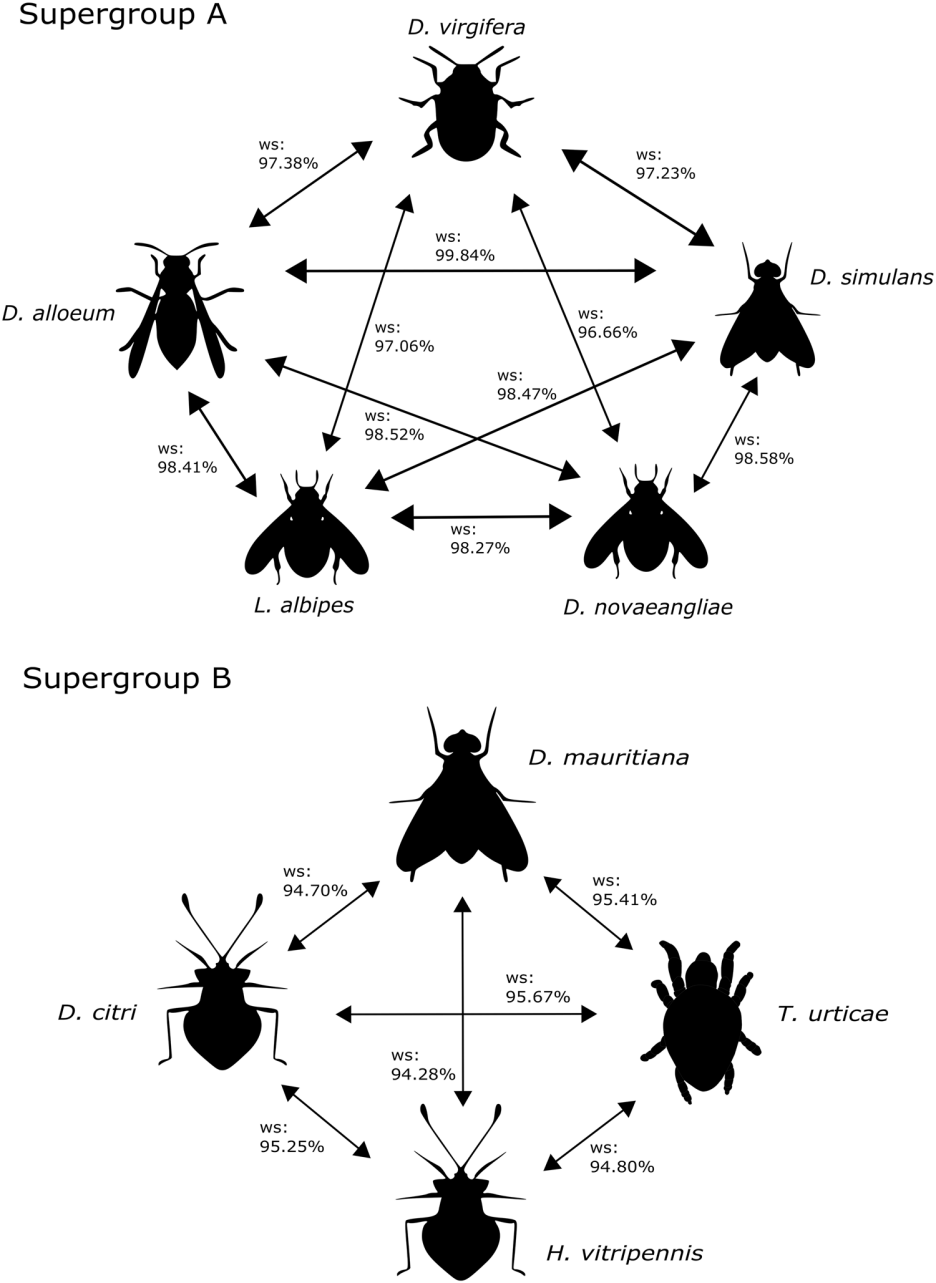
*Wolbachia* similarity between different hosts. The high *Wolbachia* similarity between distant related hosts is a strong evidence of HS since there is no feasible way of vertical transfer of *Wolbachia* between those hosts. ws, *Wolbachia* similarity.

Six out of 17 host species bearing *Wolbachia* supergroups A and B showed *Wolbachia* similarity higher than 95%, pointing out that this *Wolbachia* was shared by HS very recently, even between phylogenetically distant host taxa as Hymenoptera, Coleoptera and Diptera (Fig. 3a). Additionally, for supergroup B, four host species as phylogenetically distant as Acari, Diptera and Hemiptera share a *Wolbachia* lineage that is more than 93% similar at the nucleotide level (Fig. 3b). Therefore, from the 17 host species analysed, at least 10 (58.8%) shared *Wolbachia* lineages by HS. Thus, we ask: is HS a rare phenomenon in *Wolbachia* evolution?

HS depends on specific environmental conditions to happen, alongside the ability of a *Wolbachia* strain to infect a new host and maintain the infection^7^. It has been hypothesised that the closer the phylogenetic relationship of the hosts, the more likely HS is to occur^34^, which may induce novel phenotypes in the new host^18^. The underlying mechanisms of HS are not yet fully understood, leading it to be overlooked on many occasions.

*Wolbachia* migrates from somatic tissues to germline cells during the host’s development, transferred by cell-to-cell contact via phagocytic/endocytic machinery. Yet, in cell culture, *Wolbachia* can infect *Wolbachia*-free cells independently of cell contact through the culture medium^31^. Infection by *Wolbachia*, which is present in the haemolymph, can occur by contact with excretions or injuries of an infected host to an uninfected host^34^; thus, shared food sources and feeding habits are plausible pathways for *Wolbachia* HS between different hosts^35^. Another factor contributing to *Wolbachia* HS is predation, where ingested larvae contaminate the uninfected host, crossing the digestive system epithelium and colonising the future ovarian stem cells^36^. Parasitoid-host interactions are well documented as another route *Wolbachia* uses to move between species^12,15,18^. Among the organisms analysed in the present study, some already showed previous evidence of HS, and are either parasitoids, e.g., *Diachasma alloeum*^11^, or parasitised by a parasitoid, for example in *Drosophila melanogaster* and other *Drosophila* species^4^. HS through such interactions reinforce them as a viable mechanisms of direct *Wolbachia* transfer on a short time scale. It is important to note that, in field samples, the *Wolbachia* detected on a host may be due to sequencing reads derived from another species that are closely associated with the primary investigated host such as endoparasitoids. For instance, *Wolbachia* detected in *Ixodes ricinus*, which were actually from its endoparasitoid *Ixodiphagus hookeri*^37^, and the detection of *Wolbachia* from Strepsiptera found in the Australian tephritid fruit flies^38^. Although this may occur, it should not affect the general HS pattern identified, since there is no evidence that most of the host species analysed have endoparasitoids. Also, by the amount of data analyzed in our work and the detection of high similarity between many different species as we present here, it would be very unlikely that it is the case here, thus causing any sort of analysis bias.

The phylogenetic patterns of *Wolbachia* and its hosts usually show incongruences, indicating recent HS events and successful infection of new host species^30^. We found several instances of incongruences in the phylogenetic trees of *Wolbachia* and its hosts (Supplementary Fig. 1), reinforcing the presence of HS. Moreover, our similarity analysis showed that different *Wolbachia* show high levels of similarity within the group for both supergroup A and B (Supplementary Tables 2 and 3), whilst host similarity was lower, indicating that HS is very likely to occur in natural environments, as previously suggested^32^.

The order Coleoptera dates from more than 250 million years ago (mya), and the Diptera order around 200 mya^39^. In our analysis, the supergroup A of *Wolbachia* from both the Coleoptera *D. virgifera* and Diptera *D. melanogaster* showed very high similarity (Fig. 3a), considering that supergroup A dates from 76 mya^40^; HS presents itself as a strong hypothesis to explain the high similarity of *Wolbachia* from distantly related hosts. The same rationale is applied when comparing the Hemiptera (an order dating from nearly 350 mya) *D. citri* and *A. albopictus* (Diptera), in which their respective *Wolbachia* from supergroup B (dating from around 112 mya) also shows high similarity (Fig. 3b).

In the process of genome assembly of eukaryotic organisms, a common step is the removal of bacterial sequences. This process, although important for these studies, reduces the possibility of a proper assessment of symbionts HS^18^, which may be related to claims of HS not being a common event. In our study, using publicly available data, we calculated the within groups similarity of *Wolbachia* from supergroups A and B, tracing a parallel with their hosts’ similarity. The data showed that many *Wolbachia* from distantly related hosts share high similarity, while their hosts’ core gene similarity is significantly lower, alongside a divergence between host and *Wolbachia* phylogenetic trees. We found that 58.8% of host species analysed share two particular *Wolbachia* lineages, indicating that these lineages have been acquired by HS recently and suggesting that HS events may be more frequent than previously thought. This is evidence for the HS hypothesis being a common outcome of different ecological interactions, explaining at least partially how *Wolbachia* became such a ubiquitous organism across multiple clades. In addition, epidemiological modelling of *Wolbachia* transmission demonstrated that it would not be possible to explain *Wolbachia* incidence in a broad range of clades only considering it as vertically transmitted^41^, thus it is necessary to take host shift into account to explain the spread of *Wolbachia* in phylogenetically distant hosts.

*Wolbachia* HS is a known event described by a wide range of literature^4,6,7,14,15,32,33^, yet it is still somewhat overlooked and sometimes disbelieved as a more common mechanism^19,20,30^, as it is still not very clear how it is established in some cases^13^. Nonetheless, *Wolbachia* has an arsenal of well described methods to thrive when first encountering a new host, which may explain its success jumping across clades by HS^6^. This arsenal consists of the facts that *Wolbachia* has no problem adapting to new environments^7^, can, without much effort, move across cells and tissues, as it is a proficient manipulator of its hosts physiology^6,42^. Even though *Wolbachia* may cause reduced host fitness, the opposite is also true, as *Wolbachia* may alter pathogen susceptibility conferring viral protection for its hosts^43^. Also, *Wolbachia* can survive for a limited time in an extracellular environment, albeit being an obligatory intracellular endosymbiont^12,35^.

By using gene similarity of over 1000 reconstructed genomes^21^, alongside a phylogenetic reconstruction, we were able to bring focus to *Wolbachia* HS, estimate the event and compare it in *Wolbachia* supergroups A and B of close and distant related hosts and their *Wolbachia*, shedding more light on the importance of HS as a major player in *Wolbachia* pervasiveness on very distinctive branches of the Arthropoda tree.

## Supplementary Information

Supplementary Legends.

Supplementary Figure 1.

Supplementary Figure 2a.

Supplementary Figure 2b.

Supplementary Figure 3a.

Supplementary Figure 3b.

Supplementary Table 1.

Supplementary Table 2.

Supplementary Table 3.

Supplementary Table 4.

Supplementary Table 5.

Supplementary Table 6.

Supplementary Table 7.

Supplementary Table 8.

Supplementary Table 9.
