# Supplementary material for: Multiple long-range host shifts of major Wolbachia supergroups infecting arthropods
Source: Sci Rep. 2022 May 17;12:8131. doi: 10.1038/s41598-022-12299-x (PMC9114371; doi:10.1038/s41598-022-12299-x)
Supplement: Supplementary file 1 — Supplementary Legends. [file 41598_2022_12299_MOESM1_ESM.doc]

**Supplementary material**

**Supplementary figure 1.** *Wolbachia* phylogeny inferred using IQ-Tree and ITOL. Colors show *Wolbachia* lineages grouped by a divergence value lower than 0.02%.

**Supplementary figure 2a.** *Wolbachia* supergroup A gene alignment. Graphical representation from the concatenation of 50 *Wolbachia* orthologue genes showing high similarity between them.

**Supplementary figure 2b.** Gene alignment of *Wolbachia* hosts from supergroup A. Graphical representation from the concatenation of 50 orthologous genes of *Wolbachia* hosts showing that their similarity is lower when compared to *Wolbachia* gene similarity.

**Supplementary figure 3a.** *Wolbachia* supergroup B gene alignment. Graphical representation from the concatenation of 50 *Wolbachia* orthologue genes showing high similarity between them.

**Supplementary figure 3b.** Gene alignment of *Wolbachia* hosts from supergroup B. Graphical representation from the concatenation of 50 orthologous genes of *Wolbachia* hosts showing that their similarity is lower when compared to *Wolbachia* gene similarity.

**Supplementary table 1**. Table showing Wolbachia assembly codes, hosts and number of orthologous genes.

**Supplementary table 2**

*Supplementary table 2.1 -*Supergroup A *Wolbachia* simiilarity

*Supplementary table 2.2* **-** Supergroup A *Wolbachia* identifiers

**Supplementary table 3**

*Supplementary table 3.1 -* Supergroup B *Wolbachia* simiilarity table

*Supplementary table 2.2* **-** Supergroup A *Wolbachia* identifiers

**Supplementary table 4.1**

*Supplementary table 4.1* **-** Supergroup A and B *Wolbachia* similarity table

*Supplementary table 4.2* **-** Supergroup A and B *Wolbachia* similarity table

**Supplementary Table 5**

Descriptive statistics of *Diabrotica virgifera* and *Diachasma alloeum Wolbachia* similarities.

**Supplementary Table 6**

Descriptive statistics of *Diabrotica virgifera* and *Drosophila melanogaster Wolbachia* similarities.

**Supplementary Table 7**

Descriptive statistics of *Diabrotica virgifera* and *Dufourea novaeangliae Wolbachia* similarities.

**Supplementary Table 8**

Descriptive statistics of *Diabrotica virgifera* and *Drosophila ananassae Wolbachia* similarities.

**Supplementary Table 9**

Descriptive statistics of *Wolbachia* similarities of a supergroup B samples.
