# Supplementary material for: Multiple long-range host shifts of major Wolbachia supergroups infecting arthropods
Source: Sci Rep. 2022 May 17;12:8131. doi: 10.1038/s41598-022-12299-x (PMC9114371; doi:10.1038/s41598-022-12299-x)
Supplement: Supplementary file 2 — Supplementary Figure 1. [file 41598_2022_12299_MOESM2_ESM.pdf]

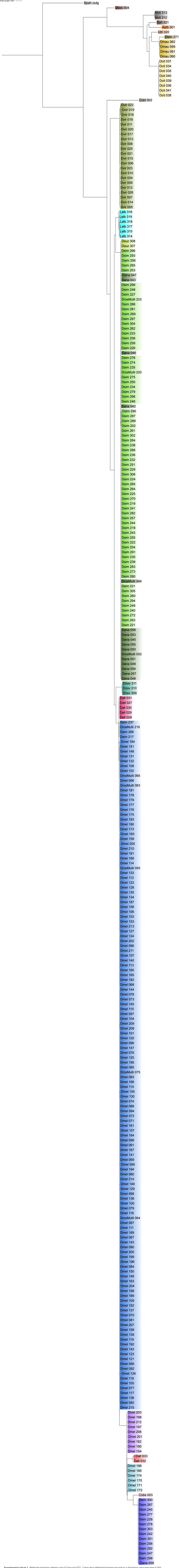

Supplementary figure 1. *Wolbachia* phylogeny inferred using IQ-Tree and ITOL. Colors show *Wolbachia* lineages grouped by a divergence value lower than 0.02%.
