# Supplementary figures and images for: Multiple long-range host shifts of major Wolbachia supergroups infecting arthropods
Source: Sci Rep. 2022 May 17;12:8131. doi: 10.1038/s41598-022-12299-x (PMC9114371; doi:10.1038/s41598-022-12299-x)

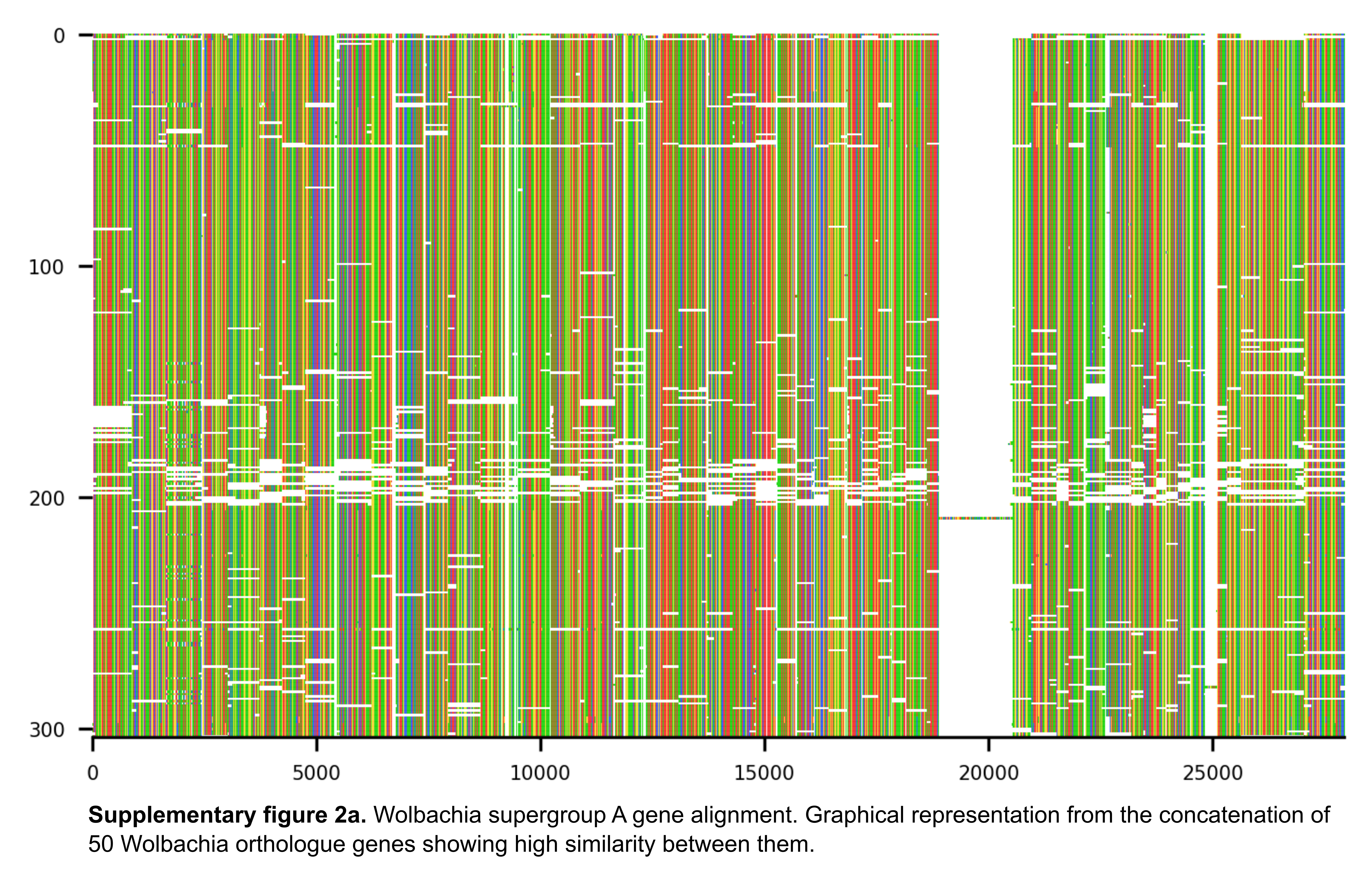

Supplement: Supplementary file 3 — Supplementary Figure 2a. [file 41598_2022_12299_MOESM3_ESM.png]

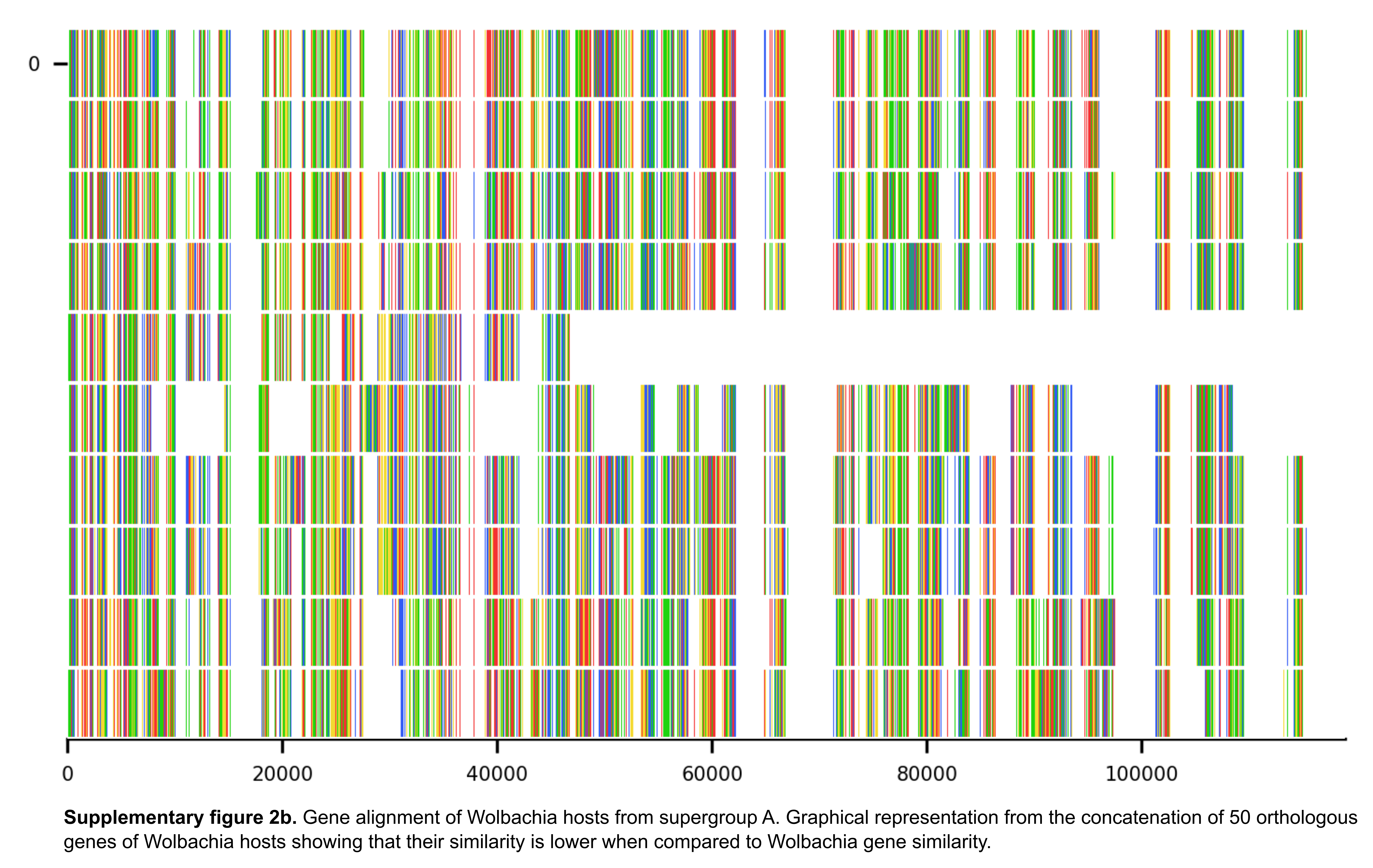

Supplement: Supplementary file 4 — Supplementary Figure 2b. [file 41598_2022_12299_MOESM4_ESM.png]

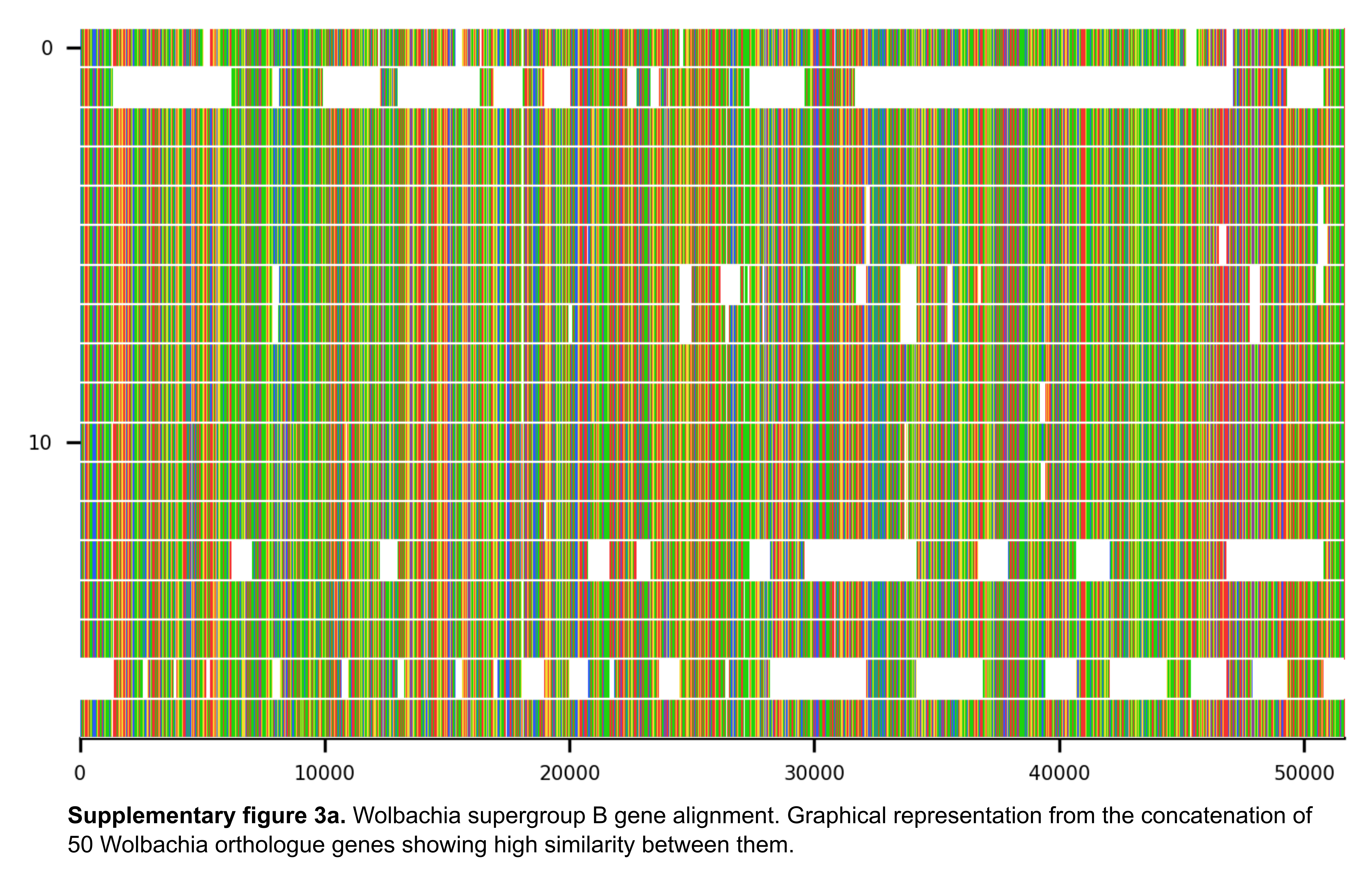

Supplement: Supplementary file 5 — Supplementary Figure 3a. [file 41598_2022_12299_MOESM5_ESM.png]

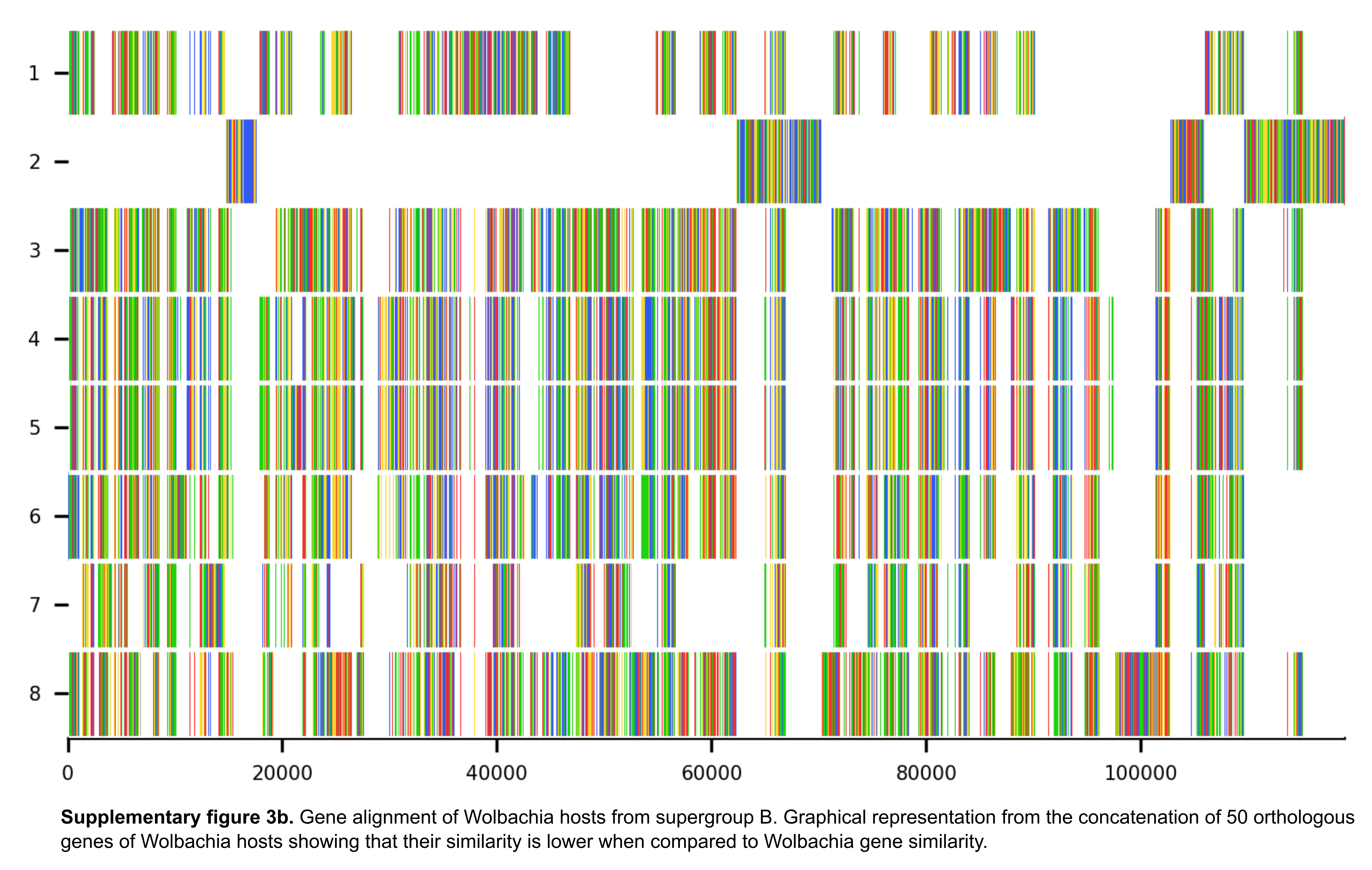

Supplement: Supplementary file 6 — Supplementary Figure 3b. [file 41598_2022_12299_MOESM6_ESM.png]
